# Construction of an immunotoxin, HN3-mPE24, targeting glypican-3 for liver cancer therapy

**DOI:** 10.18632/oncotarget.10592

**Published:** 2016-07-13

**Authors:** Chunguang Wang, Wei Gao, Mingqian Feng, Ira Pastan, Mitchell Ho

**Affiliations:** ^1^ Laboratory of Molecular Biology, Center for Cancer Research, National Cancer Institute, National Institutes of Health, Bethesda, Maryland, 20892, USA; ^2^ Department of Thoracic Surgery, Second Hospital of Jilin University, Changchun, 130041, China; ^3^ Key Laboratory of Human Functional Genomics of Jiangsu Province, Department of Cell Biology, School of Basic Medical Science, Nanjing Medical University, Nanjing, Jiangsu, 210029, China

**Keywords:** glypican-3 or GPC3, hepatocellular carcinoma, recombinant immunotoxin, single-domain antibody fragment, mouse xenograft model

## Abstract

Glypican-3 (GPC3) is overexpressed in hepatocellular carcinoma (HCC). We constructed a recombinant immunotoxin, HN3-mPE24, which contains a truncated form of Pseudomonas exotoxin A. The toxin portion lacks most of domain II and has seven point mutations in domain III to remove the B-cell epitopes thought to be responsible for causing off-target side effects and immunogenicity. We also fused a bivalent HN3 to mPE24. We tested these two molecules for GPC3 binding and cytotoxicity in HCC cell models. The *K*_D_ values of HN3-mPE24 and HN3-HN3-mPE24 for GPC3-expressing tumor cells were 12 nM and 1.4 nM, respectively. The IC_50_ values of HN3-mPE24 and HN3-HN3-mPE24 for HCC cells were 0.2 nM and 0.4 nM, respectively. We also evaluated their toxicity and anti-tumor efficacy in mice. The maximum tolerated doses of HN3-mPE24 and HN3-HN3-mPE24 were 7 mg kg^−1^ and 3.6 mg kg^−1^, respectively. We treated mice with 5 mg kg^−1^ of HN3-mPE24 intravenously every other day for ten injections. The alpha-fetoprotein level of HN3-mPE24 treated group was approximately 700 fold less than that of the untreated group (1.1 μg ml^−1^ vs. 692.1 μg ml^−1^). In addition, 25% of the mice treated with HN3-mPE24 survived to the end of this study, which was 105 days after HCC tumor implantation. In conclusion, the HN3-mPE24 immunotoxin caused liver tumor regressions and extended survival with no significant side effects in mice. It is a promising candidate for the treatment of liver cancer that may be readily translated to humans.

## INTRODUCTION

Hepatocellular carcinoma (HCC) is the most common primary liver malignancy in the world. It accounts for approximately 75% of liver cancer, with the majority of cases occurring in patients with hepatitis viral infection and chronic liver disease. Due to the geographical distribution of risk factors, the incidence of HCC is globally heterogeneous [1]. The incidence of HCC is particularly high in East Asia and sub-Saharan Africa. However, the incidence of HCC is increasing in North America and most of Europe [2]. HCC has a poor prognosis with a 5-year survival rate of 11% [3]. Most tumors are identified at an advanced stage where patients are not candidates for curative resection and the tumors are often resistant to conventional chemotherapy treatments. In early identified cases where surgery can be performed, these tumors are still associated with a 50% relapse rate [4, 5]. Sorafenib is the only FDA-approved drug for advanced HCC. This drug increases the median survival time by approximately 3 months due to the drug resistance that eventually develops [6–8]. Therefore, there is an urgent need to develop new strategies for HCC therapy.

A recombinant immunotoxin is a chimeric molecule composed of an antibody fragment fused to a toxin fragment such as Pseudomonas exotoxin A (PE). After internalization, the PE portion of the immunotoxin is separated from the antibody fragment, enters the cytosol and modifies elongation factor-2 by adenosine diphosphate (ADP)-ribosylation. This modification leads to the inhibition of protein synthesis and ultimately cell death [9, 10]. Immunotoxins are able to induce potent cytotoxicity even in cancer cells known to be resistant to standard chemotherapy. This makes them an attractive candidate for liver cancer therapy. Currently, there are several immunotoxins being evaluated clinically, including an anti-CD22 immunotoxin for drug-resistant hairy cell leukemia [11, 12] and an anti-mesothelin immunotoxin for mesothelioma [13–15]. Native PE is a 66 kDa protein consisting of three domains, in which domain III is the catalytic domain. The first generation of PE-based immunotoxins consisted of PE38, a fragment containing domain II and domain III. However, PE38 exhibited off-target toxicity and induced neutralizing antibodies in humans [16]. These drawbacks have resulted in dose limitations and reduced efficacy during clinical treatment [13]. To address these problems, a second generation PE fragment was engineered. This fragment, mPE24, has a linker containing a furin-cleavage sequence [17] instead of domain II, and domain III with seven mutations that suppress B cell epitopes [18]. These mutations have been incorporated into RG7787 that was found to have greatly reduced side effects and good anti-tumor activity when used at high doses in mice with mesothelin expressing tumors [18–21]. The potency and lack of drug resistance makes HCC immunotoxin therapy very attractive.

Glypican-3 (GPC3) is highly expressed in HCC and has been established as a histochemical diagnostic marker for liver cancer [22–24]. The expression of GPC3 has been correlated with poor prognosis in HCC [25]. GPC3 has been shown to regulate HCC cell proliferation and migration by modulating multiple signaling pathways, including Wnt, Yap [26, 27] and HGF [28]. Interestingly, surface GPC3 is efficiently internalized, which makes it an excellent candidate for immunotoxin treatment [29]. In a proof-of-concept study, we made HN3-PE38, an anti-GPC3 immunotoxin that has anti-tumor activity in HCC [29]. However, the initial immunotoxin could only be used at a relatively low dose (< 0.8 mg kg^−1^) because of side effects and *in vivo* toxicity. To develop an anti-GPC3 immunotoxin for clinical use, we decided to construct two mPE24-based immunotoxins (HN3-mPE24 and HN3-HN3-mPE24) and compared their properties in cell culture and mouse tumor models. HN3-mPE24 maintained the original antigen binding properties, showed a high level of cytotoxicity to HCC cells, and had reduced side effects in mice when given at high doses. Finally, significant tumor regression and increased survival was observed in mice with HCC tumors treated with HN3-mPE24. This data indicates that HN3-mPE24 deserves further clinical development for the treatment of HCC.

## RESULTS

### Construction of mPE24-based immunotoxins

**Figure 1:**
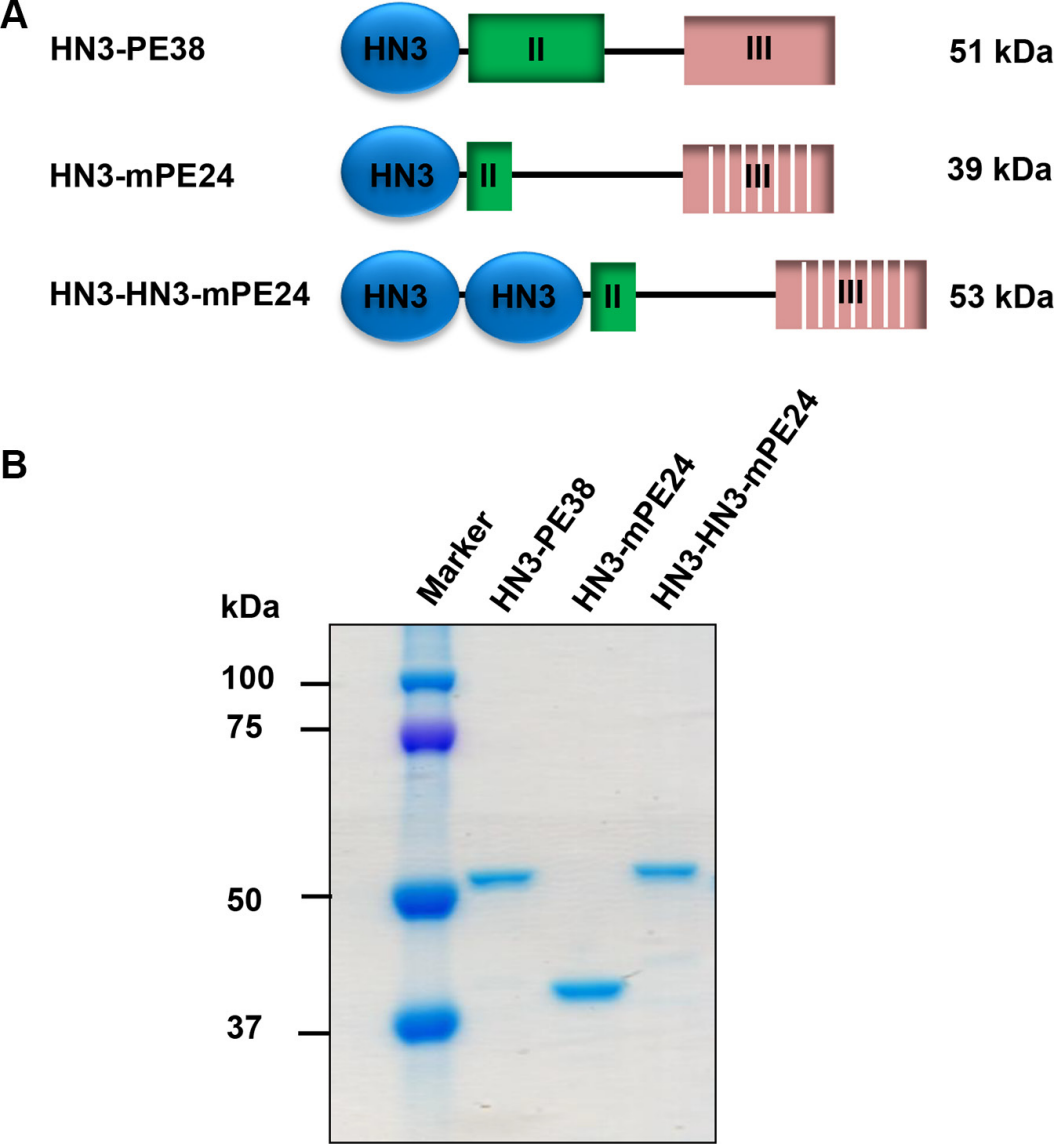
Construction of mPE24-based immunotoxins (**A**) Schematic of HN3-PE38, HN3-mPE24 and HN3-HN3-mPE24 immunotoxins. (**B**) SDS-PAGE to show the purified immunotoxins. Four micrograms of protein for each sample was loaded.

**Figure 2:**
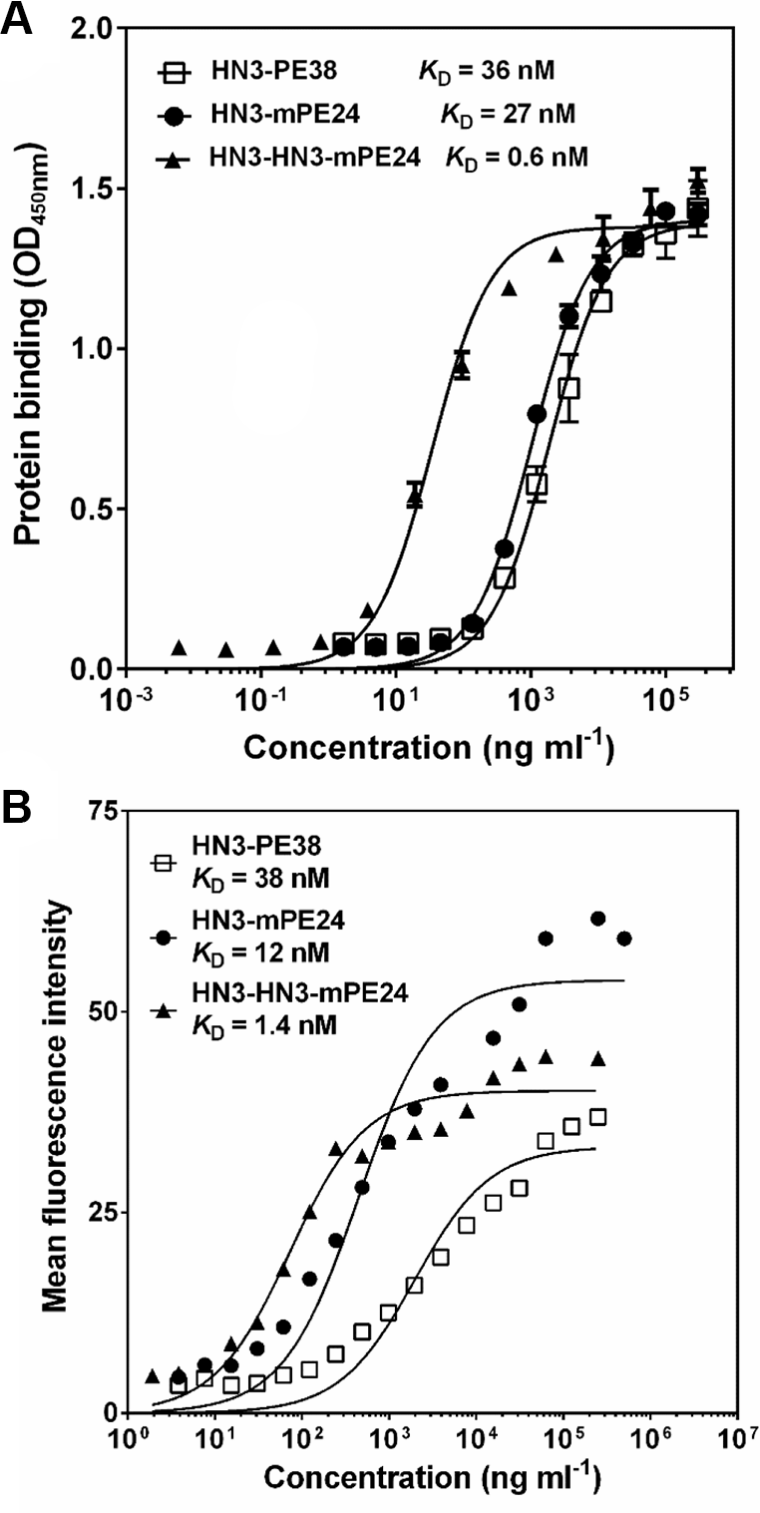
Binding properties of new immunotoxins (**A**) ELISA result showing the binding affinities of indicated immunotoxins on GPC3. (**B**) Representative flow cytometry result showed the binding affinity of indicated immunotoxins on G1 cells.

To develop an anti-GPC3 immunotoxin for clinical application, we constructed the HN3-mPE24 immunotoxin (39 kDa) using an engineered toxin fragment (mPE24) to reduce side effects and *in vivo* toxicity. We also generated a modified immunotoxin to compensate for the shortened half-life caused by the truncated PE. As shown in Figure 1, we fused bivalent HN3 to mPE24 to generate HN3-HN3-mPE24 (53 kDa). The immunotoxins were expressed in *E. coli*, refolded *in vitro*, and purified to > 90% homogeneity (Figure 1B). To evaluate the binding properties of these immunotoxins, we detected their binding to GPC3 by ELISA and flow cytometry. By ELISA, the strongest binding affinity was associated with HN3-HN3-mPE24 (*K*_D_ = 0.6 nM). HN3-mPE24 (*K*_D_ = 27 nM) was slightly better than the HN3-PE38 control immunotoxin (*K*_D_ = 36 nM) (Figure 2A). For cell binding, HN3-HN3-mPE24 showed significant improved affinity (*K*_D_ = 1.4 nM; ~30 fold); HN3-mPE24 exhibited increased affinity (*K*_D_ = 12 nM; ~3 fold) as compared to the first generation immunotoxin HN3-PE38 (*K*_D_ = 38 nM) (Figure 2B). Altogether, our results indicated that all these immunotoxins retained their ability to bind to GPC3 on cells.

### HN3-mPE24 and HN3-HN3-mPE24 retain cytotoxicity *in vitro*

**Figure 3:**
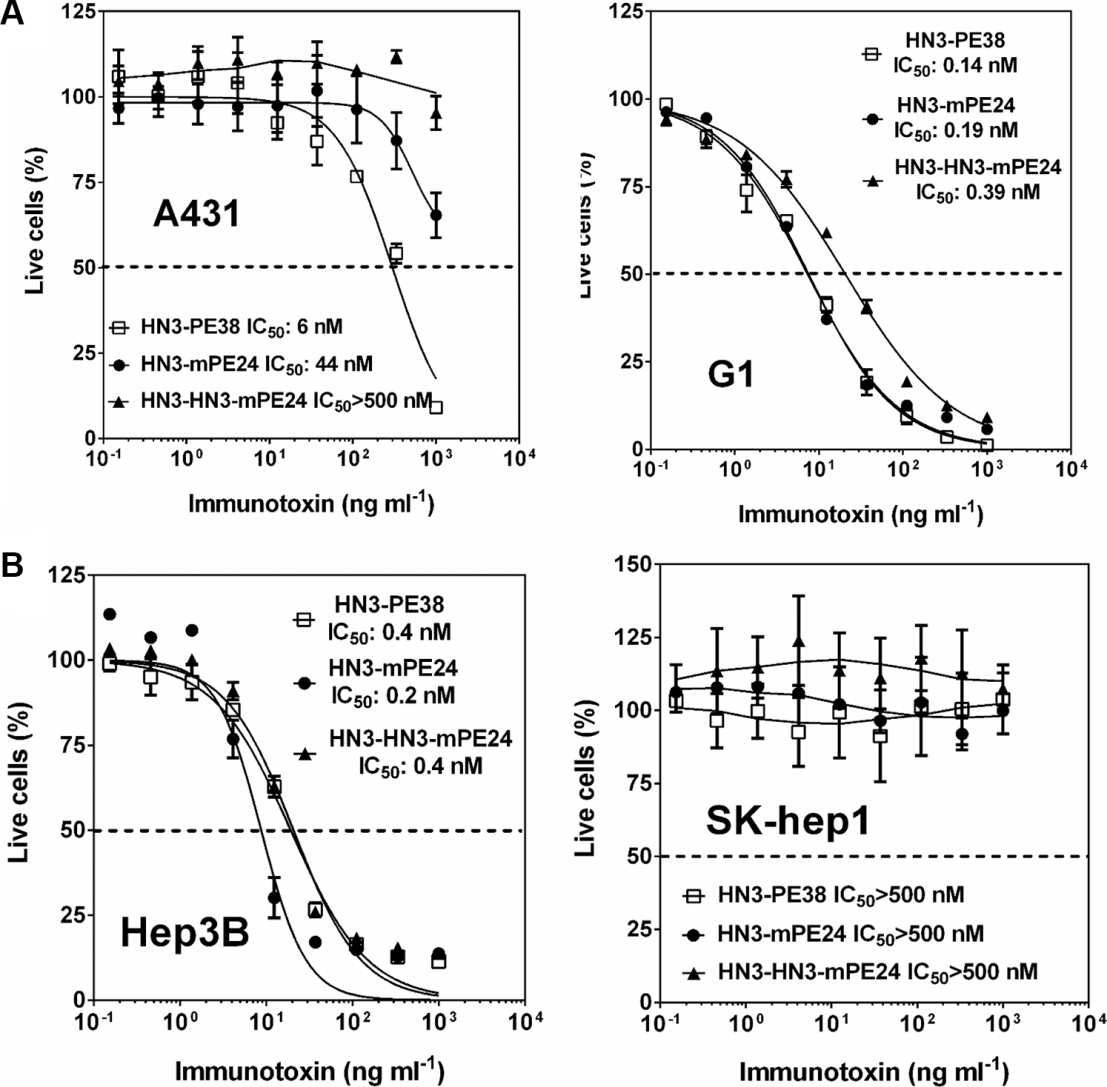
Cytotoxicity of mPE24-based immunotoxins in HCC cell culture (**A**) Inhibition of cell proliferation of A431 cells and G1 cells by indicated immunotoxins, determined by WST-8 assay. A431 was used as an antigen negative cell line. The dashed line indicates the value of IC_50_. Values represent mean ± SD. (**B**) Cytotoxicity of indicated immunotoxins on the designated HCC cells, determined by a WST-8 assay. SK-hep1 was used as an antigen negative cell line. The dash line represents 50% live cells.

To determine whether the cytotoxicity of these engineered immunotoxins correlated with their affinities, we examined the inhibition of cell proliferation on a panel of cell lines using the WST cell proliferation assay. Even though A431 cells did not express GPC3, HN3-PE38 still had a detectable cytotoxic activity at high concentrations (> 100 ng ml^−1^), indicating a nonspecific effect caused by the PE38 fragment. Interestingly, all the immunotoxins containing mPE24 had significantly reduced nonspecific cytotoxicity, suggesting that domain II may potentially contribute to nonspecific cytotoxicity (Figure 3A). We repeated the experiment with G1 cells (an A431 stable cell line overexpressing GPC3). HN3-mPE24 had similar cytotoxic activity (IC_50_ = 0.19 nM) as compared to HN3-PE38 (IC_50_ = 0.14 nM). Surprisingly, the improved affinity of HN3-HN3-mPE24 (IC_50_ = 0.39 nM) did not cause increased cytotoxicity when compared to HN3-mPE24. All of the immunotoxins had no effect on GPC3 negative SK-hep1 cells but exhibited similar cytotoxic activity on Hep3B cells (Figure 3B). Taken together, HN3-mPE24 and HN3-HN3-mPE24 had similar tumor cytotoxicity. HN3-mPE24 had significantly reduced nonspecific killing of A431 antigen negative cells.

### Mice tolerated much higher HN3-mPE24 and HN3-HN3-mPE24 doses

**Figure 4:**
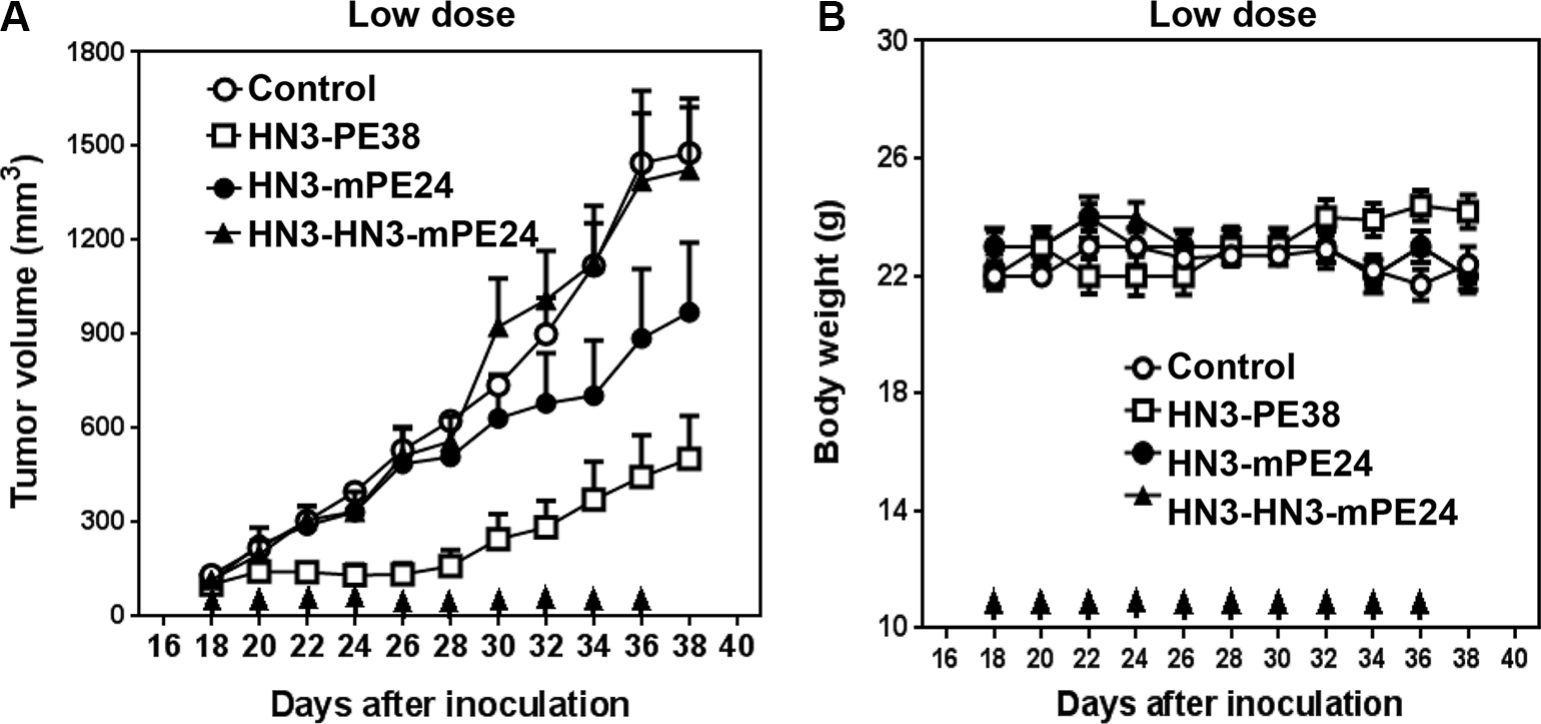
Comparison of anti-tumor activity of immunotoxins at a low dose in mice (**A**) Anti-tumor activity indicated by immunotoxins. Nude mice were s.c. inoculated with 8 × 10^6^ Hep3B cells. When tumors reached an average volume of 100 mm^3^, mice were administered indicated doses of immunotoxins intravenously (0.6 mg kg^−1^) every other day for ten injections. Arrows indicate individual injections. Control and HN3-HN3-mPE24: *n* = 8, HN3-PE38 and HN3-mPE24: *n* = 9. Values represent mean ± SE. (**B**) Body weight of the mice treated in A. Arrows indicate individual injections. Values represent mean ± SE.

**Figure 5:**
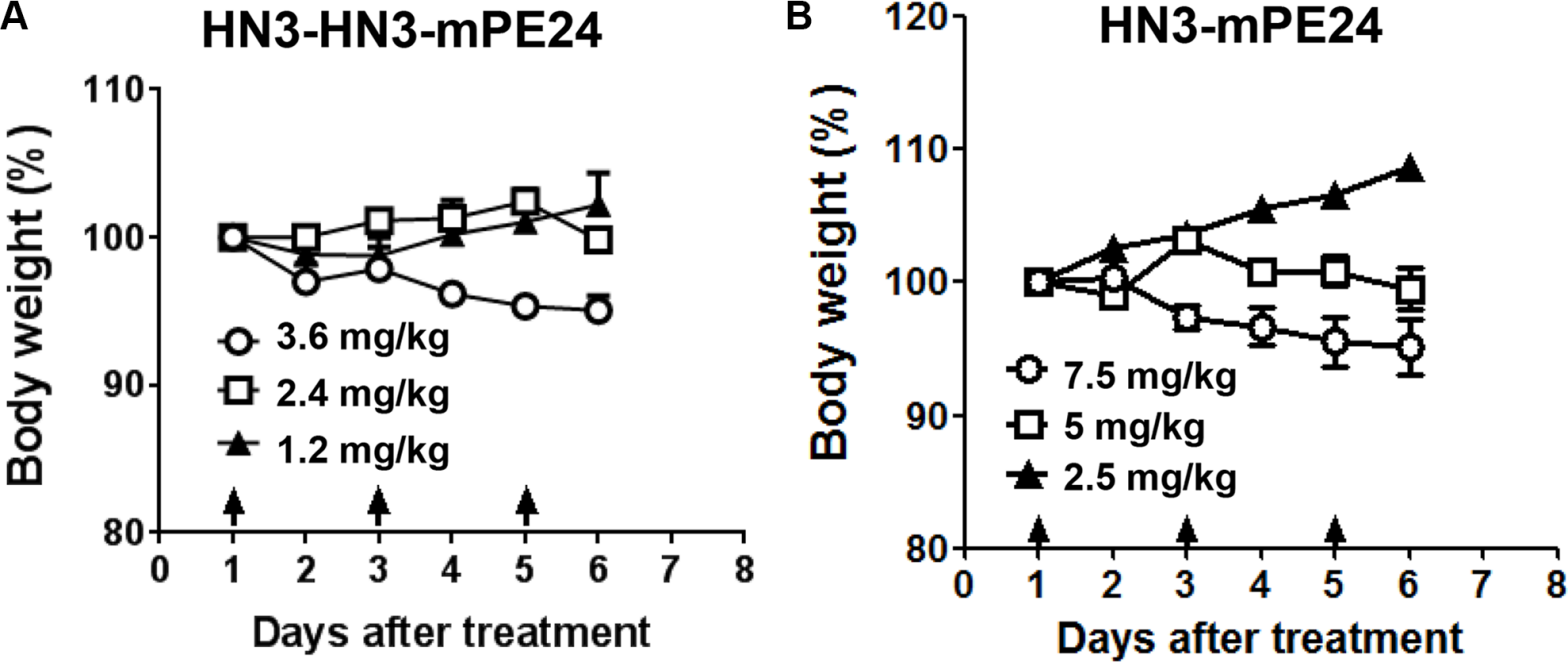
Toxicity determination of HN3-HN3-mPE24 and HN3-mPE24 in mice Nude mice were treated with the indicated dose of the HN3-HN3-mPE24 (**A**) or HN3-mPE24 (**B**) immunotoxin intravenously every other day for a total three injections. Arrows indicated individual injections. *n* = 3/group.

Our previous study showed that the mice could tolerate HN3-PE38 at a maximal dose of 0.8 mg kg^−1^; however this dose was associated with a significant loss of body weight [29]. To evaluate the anti-tumor activity of the new immunotoxins, we first treated nude mice at 0.6 mg kg^−1^, which was the maximal tolerated dose for HN3-PE38 without dramatic weight loss. The mice were treated every other day with intravenous immunotoxin for a total of 10 injections. As shown in Figure 4A, HN3-PE38 had anti-tumor activity at 0.6 mg kg^−1^. HN3-mPE24 at this concentration showed a moderate inhibition of tumor growth in the late stage of treatment. Contrary to its high affinity, HN3-HN3-mPE24 did not have any effect on tumor activity. During the treatment, no significant body weight changes were observed in any group (Figure 4B). Next, we optimized the tolerated dose of HN3-mPE24 and HN3-HN3-mPE24 in the mice without tumors. During the HN3-HN3-mPE24 treatment, the body weight of mice increased at 1.2 mg kg^−1^, remained stable at 2.4 mg kg^−1^ and decreased at 3.6 mg kg^−1^ (Figure 5A). With HN3-mPE24 treatment, the body weight of the mice increased when the dosage was kept under 5 mg kg^−1^ and remained stable at 5 mg kg^−1^. The mice started to lose weight at the 7.5 mg kg^−1^ (Figure 5B). These results indicate that, even though HN3-mPE24 and HN3-HN3-mPE24 had a lower effective anti-tumor activity when compared to HN3-PE38, the mice could tolerate a much higher dose. This was especially true for the HN3-mPE24 immunotoxin.

### HN3-mPE24 causes tumor regression in nude mice

**Figure 6:**
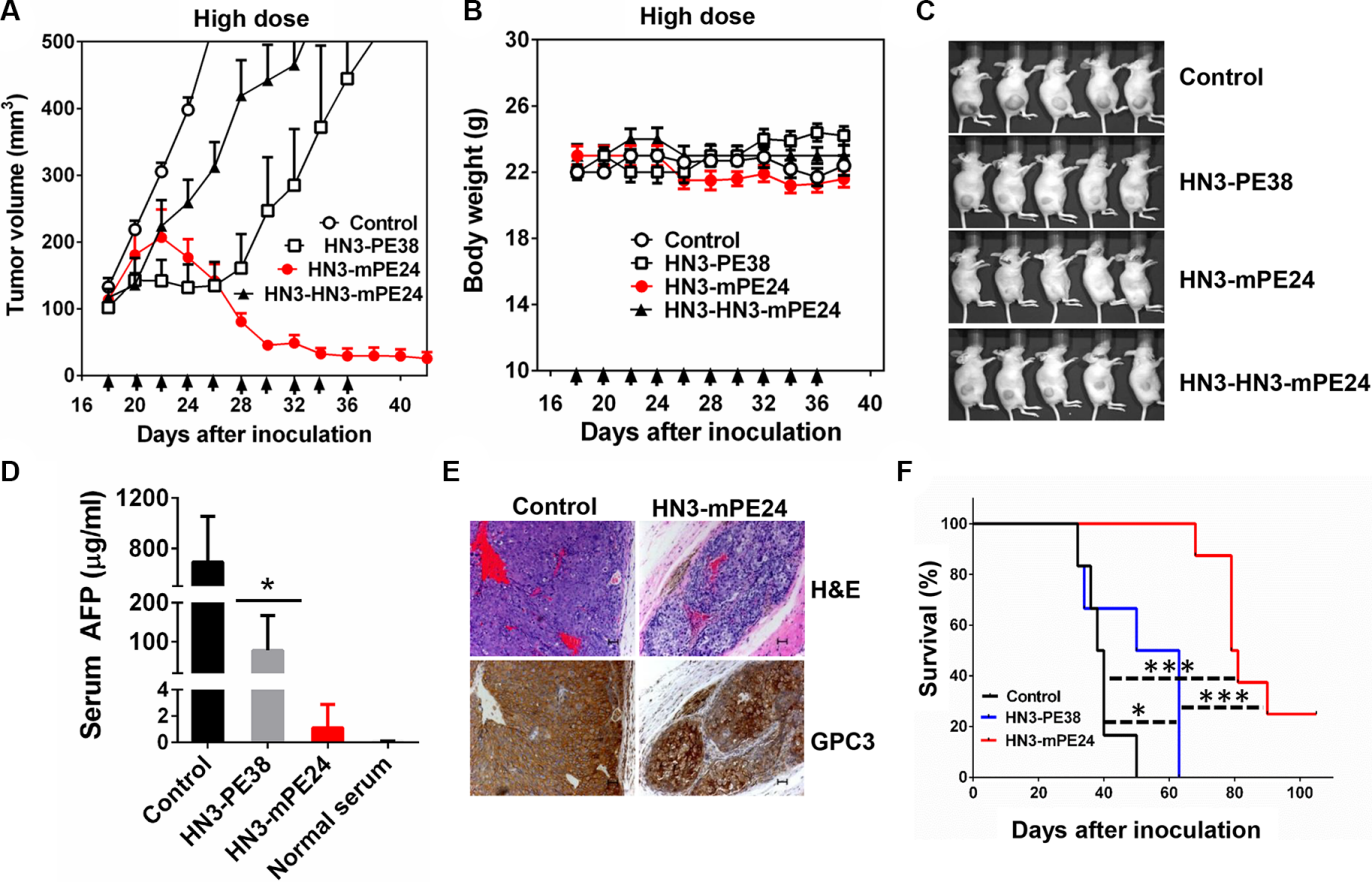
Anti-tumor activity comparison of anti-GPC3 immunotoxins at their tolerated doses in mice (**A**) Anti-tumor activity of HN3-PE38, HN3-HN3-mPE24 and HN3-mPE24 at their tolerated dose. Nude mice were s.c. inoculated with 8 × 10^6^ Hep3B cells. When tumors reached an average volume of 100 mm^3^, mice were administered HN3-PE38 (0.6 mg kg^−1^), HN3-HN3-mPE24 (2.5 mg kg^−1^) and HN3-mPE24 (5 mg kg^−1^) intravenously every other day for ten injections. Right panel shows amplified curves below 300 mm^3^. Arrows indicated individual injections. (**B**) Body weight of the mice treated in A. Arrow indicated individual injections. Values represent mean ± SE. (**C**) Representative photographs of treated mice at day 38. (**D**) Mouse serum AFP levels at day 38. Values represent mean ± SD. P* < 0.05. (**E**) H&E and immunohistochemistry staining of GPC3 on control and HN3-mPE24 treated tumor at day 38. Scale bar: 50 μm. (**F**) Survival curve for mice treated in A. *p < 0.05, ***p < 0.001 and ****p < 0.0001.

To evaluate the anti-tumor activity of HN3-mPE24 and HN3-HN3-mPE24 at their tolerated doses, we treated mice with 2.5 mg kg^−1^ HN3-HN3-mPE24 and 5 mg kg^−1^ HN3-mPE24 intravenously every other day for 10 injections. HN3-HN3-mPE24 at 2.5 mg kg^−1^ did inhibit tumor growth, but its activity was not as effective as HN3-PE38 (at 0.6 mg kg^−1^). In contrast, HN3-mPE24 led to a dramatic decrease in tumor volume (Figure 6A). The mice treated with HN3-mPE24 showed only moderate body weight loss (Figure 6B). At the end of treatment, about 70% of tumors were visually undetectable (Figure 6C). To further investigate the anti-tumor activity of HN3-mPE24, we measured the concentration of serum alpha-fetoprotein (AFP) near the end of the treatment. The AFP level of HN3-mPE24 treated group was approximately 700 fold less than that of the untreated control group and 70 fold less than HN3-PE38 treated group (1.1 μg ml^−1^ vs. 692.1 μg ml^−1^ and 68.8 μg ml^−1^, respectively) (Figure 6D). This reduction in AFP levels correlated with tumor shrinkage. We performed immunohistochemical staining on HN3-mPE24 treated tumors. The remaining tumor islets expressed GPC3 at similar levels as the untreated group (Figure 6E). This observation indicated that HN3-mPE24 treatment did not cause a down-regulation of GPC3 cell surface expression. Finally, the HN3-mPE24 treated group exhibited an impressive increase in overall survival compared to the HN3-PE38 treated group. In HN3-mPE24 treated group, 25 percent of the mice were still alive at the end of this study, which was 105 days after tumor implantation (Figure 6F). All the untreated mice died by day 50 and all the mice treated with HN3-PE38 died by day 63 after tumor inoculation.

### Toxicology of HN3-mPE24 in nude mice

In addition to measuring body weight, we also performed toxicology studies to further evaluate the side effects of HN3-mPE24 at high dose. Serum chemistry and blood cell counts in the groups treated with HN3-PE38, HN3-mPE24 and HN3-HN3-mPE24 were compared with those in the control group. We found that all groups treated with immunotoxins had an increase in white blood cells. Additionally, the HN3-PE38 and HN3-mPE24 treated groups showed an increase in alanine aminotransferase (ALT). With immunotoxin treatment, the albumin level reduced significantly compared to the untreated group, especially for HN3-mPE24 treated mice. No significant differences were detected in the other parameters measured. All organ weights in the treated mice were statistically similar to those of the control group (Table 1). Taken together, we have constructed a new HN3-mPE24 immunotoxin with reduced side effects and effective anti-tumor activity for HCC therapy.

**Table 1:** Toxicological results and organ weights

Parameters	Control	HN3-PE38	HN3-mPE24	HN3-HN3-mPE24	Normal Range 1.6–2.8
**White blood cells (K μl^−1^)**	3.92 ± 1.16	*10.7 ± 4.06	***28.57 ± 4.36	****17.11 ± 0.64	1.80–10.70
Red blood cells (M μl^−1^)	8.46 ± 0.90	8.60 ± 0.85	9.52 ± 1.15	7.22 ± 0.58	6.36–9.42
**Albumin (g dl^−1^)**	4.67 ± 0.15	**3.90 ± 0.20	***2.80 ± 0.26	**3.70 ± 0.26	1.6–2.8
**Alanine aminotransferase (U/I)**	69.33 ± 32.31	*403.67 ± 126.00	*346.67 ± 129.33	99.67 ± 47.06	29–181
Total bilirubin (mg dl^−1^)	0.37 ± 0.06	0.33 ± 0.06	0.33 ± 0.06	0.33 ± 0.06	0.0–0.6
Creatine (mg dl^−1^)	< 0.2	< 0.2	< 0.2	< 0.2	0.2–0.4
Hemoglobin (g dl^−1^)	12.33 ± 1.26	12.73 ± 0.75	13.63 ± 1.59	10.37 ± 0.81	11.00–15.10
Total protein (g dl^−1^)	5.93 ± 0.15	5.03 ± 0.25	4.2 ± 0.56	5.37 ± 0.80	4.2–5.9
Blood urea nitrogen (mg dl^−1^)	20.00 ± 2.65	19.00 ± 1.73	16.33 ± 2.51	15.33 ± 4.04	12–52
Organ Weight (mg)					
Brain	447 ± 21	440 ± 10	437 ± 38	447 ± 23	
Heart	107 ± 12	137 ± 64	113 ± 21	113 ± 12	
Kidney	287 ± 29	307 ± 35	293 ± 50	310 ± 17	
Liver	940 ± 118	*1207 ± 74	1283 ± 238	1040 ± 66	
Lung	160± 44	157± 38	200 ± 46	177 ± 32	
Spleen	137 ± 15	133 ± 15	160 ± 62	160 ± 10	

Nude mice were s.c. inoculated with 8 × 10^6^ Hep3B cells. When tumors reached an average volume of 100 mm^3^, mice were administered with 0.6 mg kg^−1^ HN3-PE38, 2.5 mg kg^−1^ HN3-HN3-mPE24 and 5 mg kg^−1^ HN3-mPE24 intravenously every other day for a total of ten injections. Blood was collected and then mice were sacrificed to obtain organs. Control and HN3-PE38: *n* = 8, HN3-HN3-mPE24: *n* = 5, HN3-mPE24: *n* = 11. Values represent mean ± SD. *p < 0.05, **p < 0.01, ***p < 0.001 and ****p < 0.0001.

## DISCUSSION

Here we constructed two mPE24-based immunotoxins against GPC3 and tested their ability to treat liver cancer in cellular and mouse models. We found that HN3-mPE24 was much less toxic to mice, with a safe dosage of 5 mg kg^−1^. Consequently, HN3-mPE24 caused HCC tumor regression and extended mouse survival significantly.

To determine which format was better, we made two different mPE24-based immunotoxins: HN3-mPE24 and a bivalent format (HN3-HN3-mPE24). We reasoned that the increase in size of HN3-HN3-mPE24 would increase the half-life when circulating in mice. The avidity of the bivalent HN3-HN3-mPE24 increased significantly in our cell binding assay. Nevertheless, HN3-HN3-mPE24 did not exhibit better anti-tumor efficacy than HN3-mPE24 in mice. This phenomenon might be attributed to better tumor penetration by the smaller size of HN3-mPE24. Our previous study demonstrated that HN3 (VH)-Fc (80 kDa) had better tumor penetration and more homogenous distribution in a xenograft tumor than YP7, a conventional human IgG antibody (150 kDa) [34]. The present study shows that the best candidate for the treatment of liver cancer is HN3-mPE24, the smallest (39 kDa) among all the immunotoxins we tested. A smaller size may facilitate efficient tumor penetration, internalization, intracellular trafficking and distribution, which all potentially contribute to better efficacy. We found that HN3-PE38 had high off-target effects on one of the antigen negative cell lines (A431), indicating the nonspecific effect caused by the PE38 fragment. The re-engineered PE fragment reduced the nonspecific cytotoxicity of immunotoxin on A431 cells and showed reduced toxicity and side effects in mice. This result was consistent with previous reports showing that the immunotoxin against mesothelin exhibits a significantly reduced side effects upon removal of domain II of native PE [19, 20]. Although HN3-PE38 showed anti-tumor activity at doses as low as 0.6 mg kg^−1^, it requires re-engineering for clinical applications because of the high level of nonspecific cytotoxicity associated with higher doses. The reduction of off-target effects elevated the tolerated dose of HN3-mPE24 in mice to as much as 5 mg kg^−1^. This is approximately 10 fold higher than that of HN3-PE38 [29]. We noticed that HN3-PE38 and HN3-mPE24 treated mice showed increased ALT levels. Elevated ALT may indicate liver damage. However, we did not note any gross evidence of liver damage in mouse necropsy. Transient elevation of ALT has been observed in patients treated with other immunotoxins [35]. The elevation of ALT was reversible in patients. Increase of ALT in patients normally occurred within 24 h after the immunotoxin treatment. After the treatment stopped, ALT levels went down to the normal range in about a week. Further preclinical studies would be needed to validate the effect of HN3-mPE24 systematically, including its pharmacodynamics, pharmacokinetics and influence on liver function. HCC has proven to be unresponsive to most chemotherapy drugs [36]. Sorafenib is the only drug approved for advanced HCC; however HCC tumor cells develop resistance to sorafenib treatment by modulating many signaling pathways [8], including PI3K/Akt [37] and EGFR/Her-3 [38]. In our study, HN3-mPE24 treated tumors exhibited strong staining for GPC3 at the end of treatment, indicating no loss of antigen expression after HN3-mPE24 treatment. This suggests that further regression could be achieved through optimization of immunotoxin treatment. Several previous studies have shown that continuous infusions of immunotoxin significantly improve anti-tumor activity [39, 40]. Immunotoxins overall possess short half-lives [32, 41, 42], therefore, a low dose, high frequency treatment might be the most effective approach. HN3-mPE24's ability to penetrate tumors makes it suitable to use as a follow up treatment after radiotherapy, chemotherapy and surgery. Other strategies to increase HN3-mPE24 efficiency include silencing of T cell epitopes to further decrease immunogenicity [43] and combining it with chemotherapy or immune suppression to allow as many treatment cycles as possible [15]. These will be evaluated in future preclinical and clinical studies.

In conclusion, our results demonstrate that the new HN3-mPE24 immunotoxin causes regression of HCC xenografts in mice with improved efficacy and no significant side effects. These data strongly support HN3-mPE24 as a promising candidate for the treatment of liver cancer in humans.

## MATERIALS AND METHODS

### Cell culture

A431 and Hep3B cell lines were purchased from American Type Culture Collection (Manassas, VA). G1, a transfected A431 cell line stably expressing human GPC3, was generated in our lab [30]. SK-hep1 was a kind gift from Dr. Xin-Wei Wang at the National Cancer Institute (Bethesda, MD). SK-hep1 was originally isolated from a patient with adenocarcinoma of the liver but was redefined as a non-HCC line [31]. The cell lines were cultured in DMEM supplemented with 10% fetal bovine serum, 100 U ml^−1^ penicillin, 0.1 mg ml^−1^ streptomycin, and 2 mmol l^−1^ L-glutamine. All cell lines were passaged less than 15 times at the time of usage. All cell lines were tested and authenticated by morphology and growth rate and were found to be mycoplasma free.

### Production of a recombinant immunotoxin

The HN3 human single domain VH was isolated using phage display technology in our laboratory [32]. The synthetic HN3-mPE24 (pMH212) and HN3-HN3-mPE24 (pMH213) fragments were cloned into the pRB98 vector by NdeI and EcoRI restriction sites to produce the indicated plasmids. The cloning procedures were described in our laboratory protocol for immunotoxin production [33]. The HN3-PE38 plasmid (pMH150) had been produced previously in the lab [29].

### ELISA

Different immunotoxins (50 μl/well) were added to 96-well ELISA plates coated with 5 μg ml^−1^ of GPC3-hFc, and incubated at room temperature for 1 hour. Plates were then washed five times with PBST (0.5% Tween 20 in PBS) followed by incubation at room temperature for 1 hour with 50 μl of 1:200 dilution of rabbit anti-Pseudomonas exotoxin A exotoxin antibody (Sigma, St. Louis, MO). The plates were then washed five times with PBST followed by incubation at room temperature for 1 hour with 50 μl of goat anti-rabbit HRP conjugate at 1:5000 dilution (Jackson Laboratory, Bar Harbor, ME). After washing three times with PBST, 50 μl/well of 3,3′,5,5′-tetramethylbenzidine detection reagent (KPL, Gaithersburg, MD) was added, and the plate was incubated for 10 minutes at room temperature. Substrate development was stopped by the addition of 0.1 N sulfuric acid. Absorbance was read at 450 nm.

### Flow cytometry

Single cell suspensions of G1 cells were incubated with different concentrations of immunotoxins for 1 hour on ice and then incubated with a 1:200 dilution of rabbit anti- Pseudomonas exotoxin (Sigma) for 1 hour on ice. Bound antibodies were detected by incubating with goat anti-rabbit IgG-PE at 1:200 (Invitrogen, Camarillo, CA) in FACS buffer (PBS, 5% BSA) for half an hour on ice. Cells were analyzed using FACS Calibur (BD Biosciences, San Jose, CA).

### Cell proliferation assay

Cells were seeded into 96-well plates at a concentration of 10^4^ cells per well. After overnight culture, different concentrations of immunotoxins were added into the wells. Cell growth inhibition was measured by WST-8 (Dojindo Molecular Technologies, Rockville, MD) assays 72 hours later. The cytotoxicity was presented as IC_50_, which is the toxin concentration that reduced cell proliferation by 50% compared with cells that were not treated with the toxin.

### Animal studies

All mice were housed and treated under the protocol approved by the Institutional Animal Care and Use Committee (IACUC) at the National Institutes of Health (NIH). Hep3B cells (8 × 10^6^) were suspended in 200 μl of PBS and inoculated subcutaneously (s.c.) into 5 week-old female athymic NCr-nu/nu nude mice (NCI- Frederick Animal Production Area, Frederick, MD). Tumor dimensions were determined using calipers and tumor volume (mm^3^) was calculated by the formula V = ab²/2, where a and b represent tumor length and width, respectively. When the average tumor size reached approximately 100 mm^3^, the mice were intravenously injected with the indicated doses of immunotoxins every other day. Mice were euthanized when the tumor size reached 1500 mm^3^.

### Detection of serum alpha-fetoprotein (AFP)

Serum AFP levels were determined by ELISA using an Enzyme Immunoassay kit (GenWay Biotech, Inc, San Diego, CA). Whole blood samples were collected from each group at the end of treatment. The concentration of AFP in the serum was measured according to the manufacturer's instructions.

### Toxicological analysis

Three nude mice from each drug treatment group were chosen for toxicology studies. Samples were processed for completed blood counts (CBC), comprehensive serum chemistry (VetScan, Abaxis Veterinary Diagnostics, Union City, CA) and internal organ weights. These analyses were performed by the Pathology/Histotechnology Laboratory in NCI-Frederick, MD.

### Statistical analysis

All the reported results were repeated in at least three independent experiments. All group data (except those indicated) were expressed as the mean ± standard deviation (s.d.) of a representative experiment performed in at least triplicate and similar results were obtained in at least three independent experiments. All statistical analyses were conducted using GraphPad Prism 6.0 (GraphPad Software, Inc., La Jolla, CA). Differences between groups were analyzed using the two-tailed Student *t* test of means, with P* < 0.05 defined as significant.

## Abbreviations

HCC: hepatocellular carcinoma
PE: Pseudomonas exotoxin A
ADP: adenosine diphosphate
GPC3: Glypican-3
s.c.: subcutaneously
AFP: alpha-fetoprotein
